# Long-term nitrogen addition affects the phylogenetic turnover of soil microbial community responding to moisture pulse

**DOI:** 10.1038/s41598-017-17736-w

**Published:** 2017-12-13

**Authors:** Chi Liu, Minjie Yao, James C. Stegen, Junpeng Rui, Jiabao Li, Xiangzhen Li

**Affiliations:** 1 Key Laboratory of Environmental and Applied Microbiology, Chinese Academy of Sciences; Environmental Microbiology Key Laboratory of Sichuan Province, Chengdu Institute of Biology, Chinese Academy of Sciences, Sichuan, 610041 China; 2 Earth and Biological Sciences Directorate, Biological Sciences Division, Pacific Northwest National Laboratory, Richland, WA 99352 USA; 3 Fujian Provincial Key Laboratory of Soil Environmental Health and Regulation, College of Resources and Environment, Fujian Agriculture and Forestry University, Fuzhou, 350002 China

## Abstract

How press disturbance (long-term) influences the phylogenetic turnover of soil microbial communities responding to pulse disturbances (short-term) is not fully known. Understanding the complex connections between the history of environmental conditions, assembly processes and microbial community dynamics is necessary to predict microbial response to perturbation. We started by investigating phylogenetic spatial turnover (based on DNA) of soil prokaryotic communities after long-term nitrogen (N) deposition and temporal turnover (based on RNA) of communities responding to pulse by conducting short-term rewetting experiments. The results showed that moderate N addition increased ecological stochasticity and phylogenetic diversity. In contrast, high N addition slightly increased homogeneous selection and decreased phylogenetic diversity. Examining the system with higher phylogenetic resolution revealed a moderate contribution of variable selection across the whole N gradient. The moisture pulse experiment showed that high N soils had higher rates of phylogenetic turnover across short phylogenetic distances and significant changes in community compositions through time. Long-term N input history influenced spatial turnover of microbial communities, but the dominant community assembly mechanisms differed across different N deposition gradients. We further revealed an interaction between press and pulse disturbances whereby deterministic processes were particularly important following pulse disturbances in high N soils.

## Introduction

The processes shaping the composition and structure of communities are central topics in ecological research. Microbial communities are important to maintaining crucial soil functions^1^. Soil microbial communities are highly diverse, and they can change rapidly in compositions at different timescales^2,3^. In addition, because of highly diversified physiologic and genomic properties of microorganisms, it is difficult to understand the detailed processes shaping soil microbial community structure under different environments. So far, it is a formidable challenge to construct a framework to systematically understand the relationships among phylogeny, function, diversity, and environment^2,4,5^. Understanding the factors that generate and maintain soil microbial diversity and assemblages holds special significance for ecologists.

Disturbances are causal events^6^ and are often classified as pulses or presses depending on their duration^7^. In general, pulse disturbances are relatively discrete, short-term events that are often intense and can rapidly decrease in severity, whereas presses are long-term or continuous events that may arise sharply but reach a constant level that is maintained over a long period of time^8^. Pulses and presses may significantly impact microbial community structure and assembly by affecting microorganisms directly and/or the reallocation of resources^9^. From the viewpoint of population, the response of microorganisms to disturbance is often determined by physiological mechanisms^10^ and/or ecological mechanisms associated with adaptation and competition^11^.

Soil microbial communities are structured by both historical factors (e.g., speciation and large-scale dispersal) and contemporary processes (including niche-based and neutral-based processes)^4,12^. Null models have often been used to disentangle deterministic (abiotic and biotic filtering) versus stochastic (e.g., drift and dispersal) effects on community assembly^13–15^. Recent studies that work to decipher community assembly processes and the factors that impose them often leverage phylogenetic approaches combined with null models^16–20^. Although the importance of phylogenetic structure and turnover has been acknowledged in microbial community ecology, limited information is available about the relationships among different disturbances, phylogenetic structure, and community turnover.

By extending Vellend’s theoretical framework^21^ at the scale of a metacommunity, Stegen *et al*.^19^ propose a comprehensive null modeling approach that estimates the relative influences of ecological processes on community assembly. Ecological selection results from different organisms having different levels of fitness for a given set of ‘environmental conditions’ (abiotic variables and biotic factors related to organismal interactions). If environmental conditions are homogeneous through space or time, the selective environment is also homogeneous^21^. The selection caused by the consistent selective pressure in homogeneous environment is defined as ‘homogeneous selection’. Homogeneous selection usually causes low compositional turnover. Selection caused by changes in selective pressure through space or time is called ‘variable selection’. Variable selection usually causes high compositional turnover. When selection is not the primary cause of compositional turnover, low levels of dispersal coupled with ecological drift can result in large differences in community composition; this scenario is referred to as dispersal limitation. On the other hand, very high rates of dispersal can homogenize community composition; this is referred to as homogenizing dispersal. Cases in which no single process strongly drives observed differences in community composition are referred to as ‘undominated’^19^.

Large shifts are observed in microbial community compositions along N addition gradients^22,23^. An important reason is that long-term N deposition can lower soil pH and increase NH_4_^+^-N and salinity, resulting in significant changes to the selective environment^23^. Moisture pulse is also known to have a large influence on microbial community structure^24^. However, it is not clear to what extent the spatial phylogenetic turnover shifts along N addition gradients, and whether the N addition history alters the microbial response to moisture pulse.

**Figure 1:**
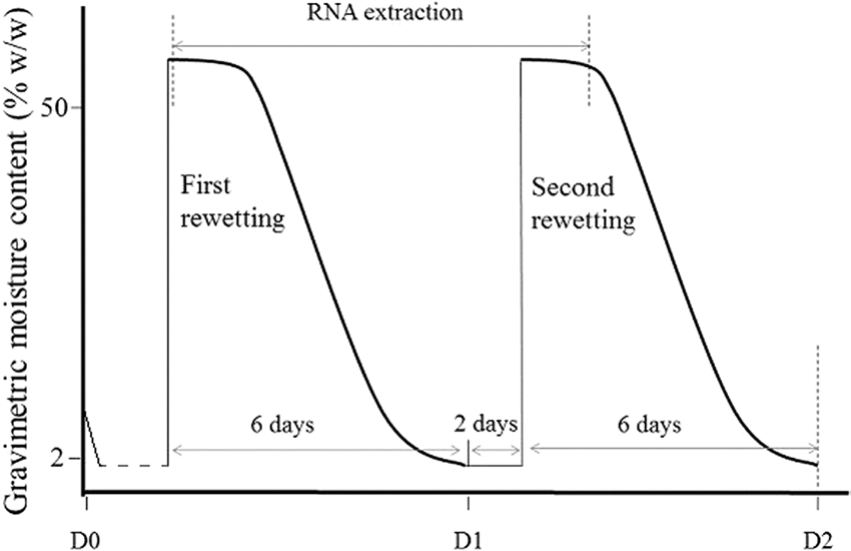
Schematic of the experimental design showing the drying and wetting treatments. D0 denoted the time point for field soil sampling; D1 was the time point after the first wetting and drying cycle. A pre-experiment had been conducted to determine the total incubation time.

In this study, press disturbance was defined as long-term N (NH_4_NO_3_) addition, and pulse disturbance as rewetting and rapid drying. We used these two disturbances to fill the knowledge gaps summarized above. We collected soil samples from a 13-year field experiment with different N deposition rates. The phylogenetic turnover of microbial communities along the N gradient was explored based on 16S rDNA amplicon sequencing. We used ‘press’ to specifically represent the long-term annual effects of N addition, which may cause species sorting across the whole gradient or the acclimation of some microbes to the altered conditions. In many ecosystems, stochasticity in community assembly can increase with resource supply^13,25^. We therefore predict that there may be an increase of the relative importance of stochastic processes with elevated N deposition rates. On the other hand, strong, niche-based processes are often found in harsh environments for both micro- and macro-organism systems^26–28^. If N addition increases the strength of ecological selection through, for example, increases in salinity, communities from the highest N soils may be characterized by the strongest influences of deterministic processes. We also designed a continuous drying-rewetting experiment (Fig. 1) to investigate the turnover of the prokaryotic community based on 16S rRNA, which has been commonly used as a biomarker to study microbial activity potential and succession^24,29,30^. Using rRNA instead of rDNA in the moisture pulse experiment facilitates the detection of sensitive responses of microbes to environmental fluctuations at the ribosome expression level. We expect that the response to pulse disturbance will depend on N addition history, and that the rate of phylogenetic turnover will increase with the strength of deterministic assembly under press disturbance.

## Results

### Phylogenetic signal detection

Inferring ecological mechanisms using phylogenetic turnover requires phylogenetic signals in organismal niches^16,31^. We therefore evaluated phylogenetic signals to reveal the extent to which closely related taxa have more similar niche preferences than distant relatives^31^. Two methods were used for detecting phylogenetic signals. Pagel’s λ^32^ is based on an evolutionary drift (Brownian motion) model and has been shown to perform well for detecting phylogenetic signal^33^. The P-value for λ is calculated with a likelihood ratio test, where the observed λ is compared to a trait distribution without phylogenetic signal. We also performed Mantel correlograms, which are useful for characterizing the continuous correlations between niche values and phylogenetic distance^18,34^.

**Figure 2:**
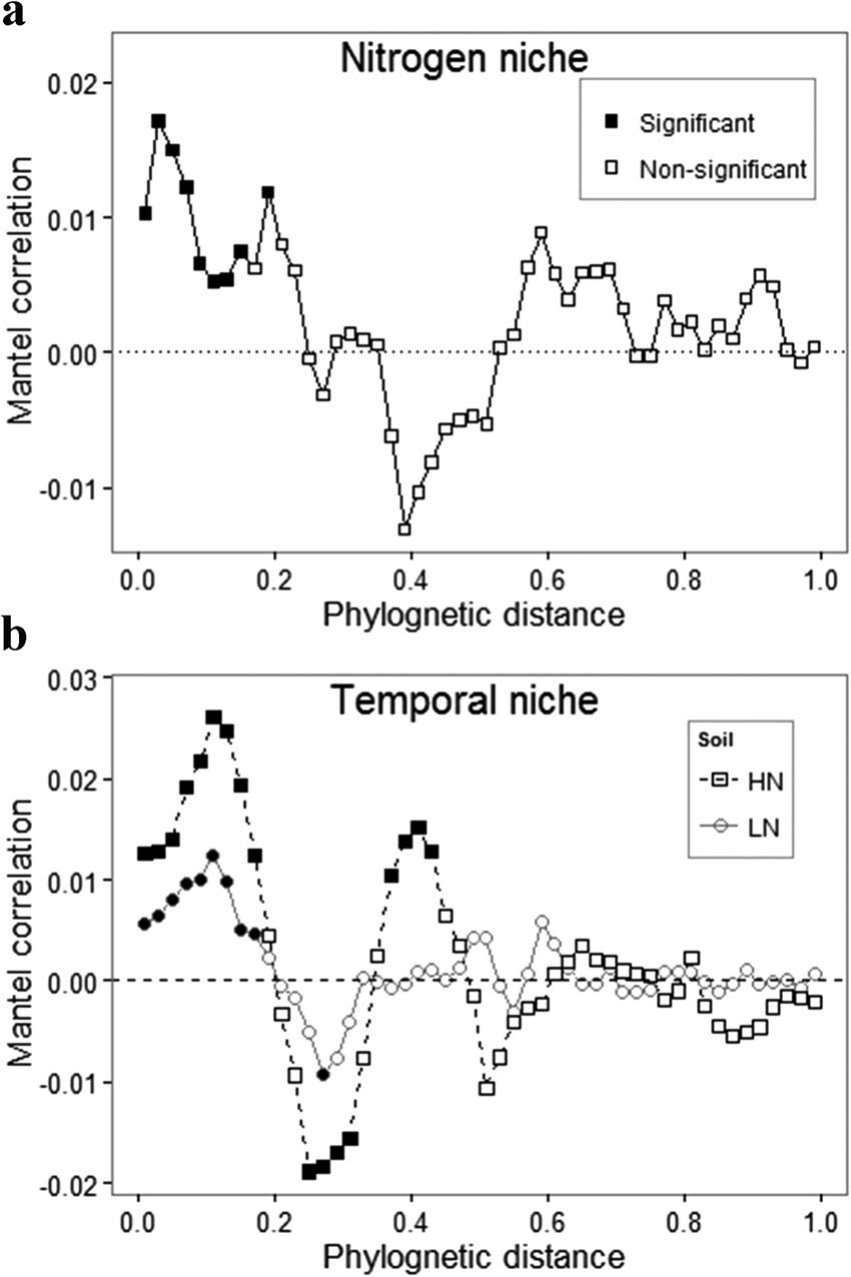
Mantel correlogram (**a**: nitrogen niche; **b**: temporal niche) between the Euclidean distance matrix of OTU niche values and phylogenetic distance matrix. Solid symbols denoted significant (*P* < 0.05, 1000 permutations) correlations (phylogenetic signal), and open symbols denoted non-significant points. The OTU niche values were estimated using abundance-weighted approach. The phylogenetic distance was obtained by transforming phylogenetic tree into Patristic distance and partitioned into classes by 0.02 units for calculations. LN: low nitrogen soil (N deposition rate: 5.25 g N m^−2^ yr^−1^); HN: high nitrogen soil (28 g N m^−2^ yr^−1^).

Significant phylogenetic signals (*P* < 0.0001) were observed in both the N niche and temporal niche based on Pagel’s λ (for N, λ = 0.848, and for time, LN, λ = 0.474; HN, λ = 0.79). Mantel correlograms showed significant phylogenetic signals across relatively short phylogenetic distances, which disappeared at about 20% of the maximum phylogenetic distance for both the N and temporal niches (Fig. 2). Both approaches therefore indicated significant phylogenetic signals, thereby enabling use of phylogenetic turnover patterns to infer ecological processes.

### Phylogenetic diversity in the N deposition soils

**Figure 3:**
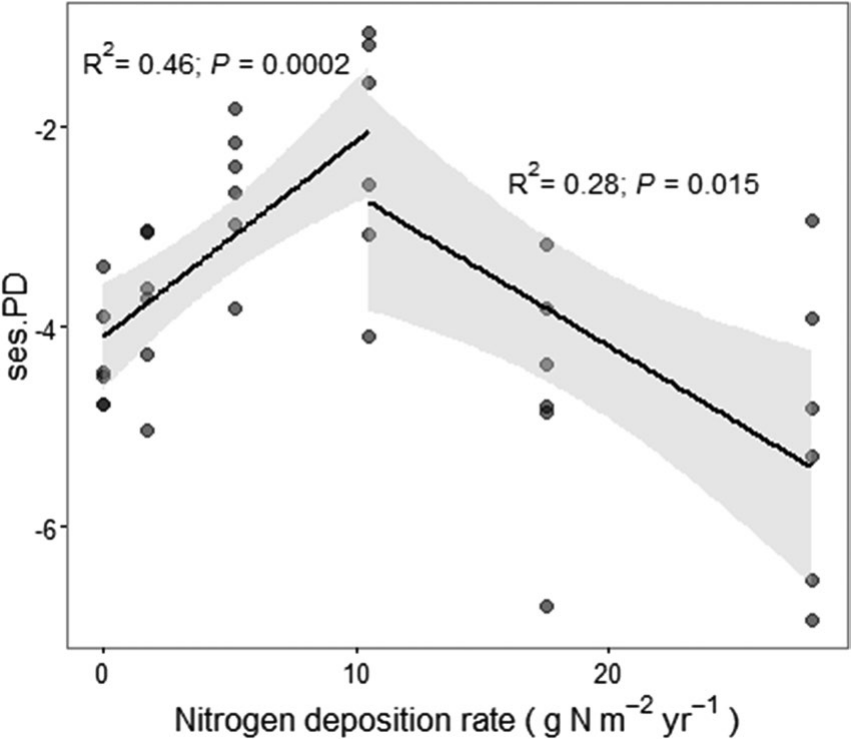
The shifts of standardized effect size of phylogenetic diversity (ses.PD) with the increase of N deposition rate (0, 1.75, 5.25, 10.5, 17.5, 28 g N m ^−2^ yr^−1^). The segmented linear regressions were fitted according to two N ranges, 0–10.5 and 10.5–28 g N m^−2^ yr^−1^. The corresponding statistics were provided. Grey bands represented the bootstrapped 95% confidence intervals. There were 36 samples in total, and 6 samples at each N level. We used semi-transparent black points instead of the pure black points to avoid the confusion about the number of samples.

To explore the long-term effect of N addition on the species pool along the N gradient, we calculated phylogenetic diversity^35^ and the standardized effect size (ses.PD)^36,37^. Regression analysis showed a positive relationship between ses.PD and the N deposition rate range from 0 to 10.5 g N m^−2^ yr^−1^ (R^2^ = 0.46, *P = *0.0002; Fig. 3), and a negative relationship between ses.PD and the N deposition rate range from 10.5 to 28 g N m^−2^ yr^−1^ (R^2^ = 0.28, *P* = 0.015). Therefore, there were different patterns of phylogenetic diversity under different intensities of environmental pressures imposed by N addition.

### Phylogenetic turnover in the N deposition soils

**Figure 4:**
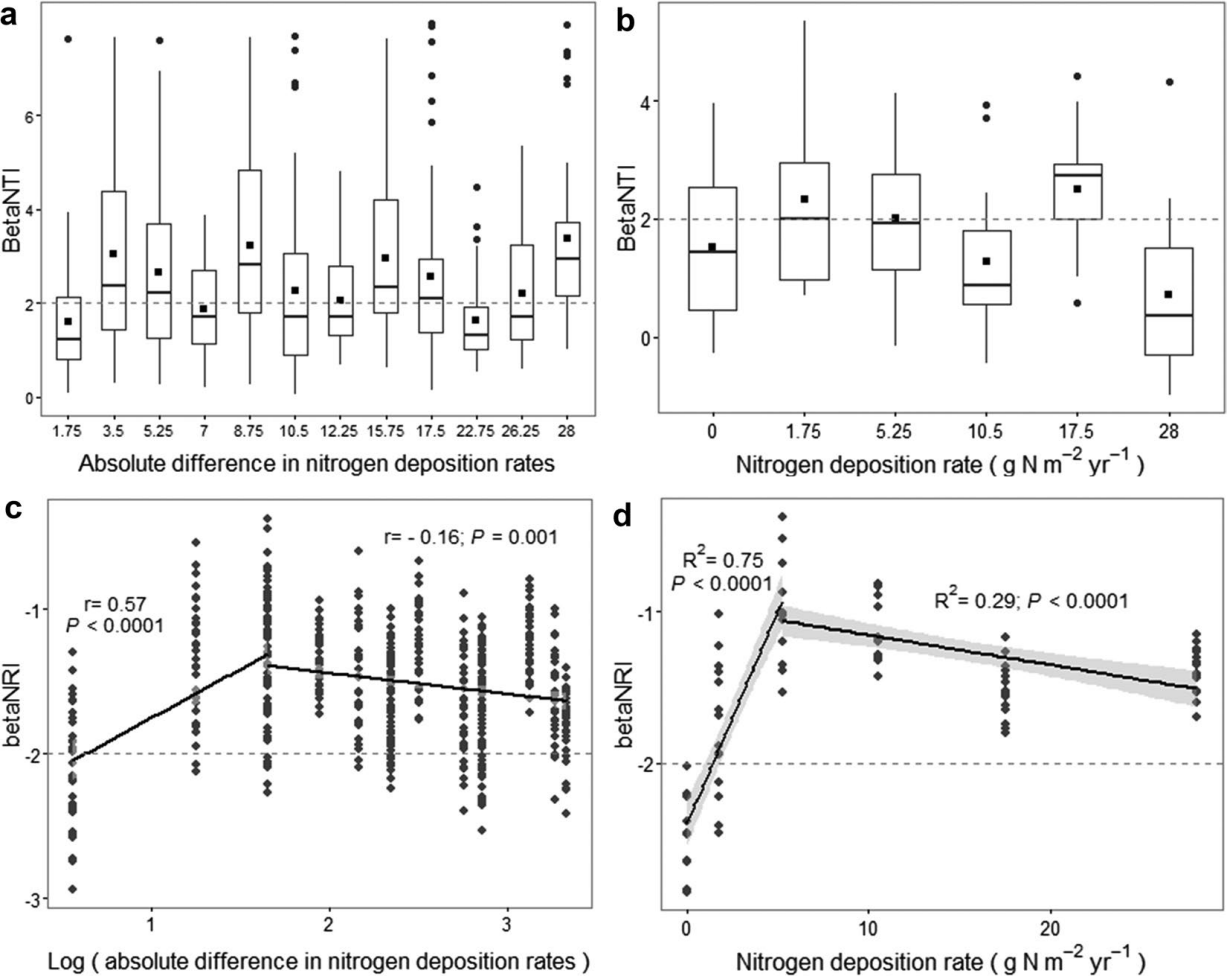
Patterns of βNTI (**a**,**b**) and βNRI (**c**,**d**) in N deposition experiments. Boxplot was used to show βNTI patterns for all pairwise community comparisons between N levels (**a**) and within N levels (**b**). Boxes represented the median and 25th/75th percentile and the solid lines extended to 1.5 times the interquartile range. βNRI values were fitted against Ln-transformed absolute differences in N deposition rates (between N levels; **c**) and the raw N deposition rates (within N levels; **d**) using segmented regression approach. The corresponding statistics were provided in each panel. Horizontal dashed lines denoted values of +2 or −2, and the values beyond which an individual β-deviation value was considered statistically significant. Grey bands represented the bootstrapped 95% confidence intervals.

To evaluate phylogenetic turnover along N gradient between N levels and within each N level, beta nearest taxon index (βNTI) and beta net relatedness index (βNRI) were both used to explore mechanisms underlying community assembly^38,39^. For pairwise comparisons of βNTI and βNRI, we inferred homogeneous selection if values were <−2 and variable selection if values were >+ 2 (approximate values of ± 1.96 at two-sided 5% error level of each null distribution). If −2 < values <+2, stochasticity (e.g., dispersal and drift) may have a stronger influence than deterministic selection^18,19^. The analysis of βNTI revealed a moderate level of variable selection that was observed along the whole N gradient (Fig. 4a and b). We did not find a significant relationship between βNTI and sample-to-sample pairwise differences (Ln-transformed absolute difference) at different N levels (Fig. 4a) or within each N level (Fig. 4b). In contrast, for pairwise comparison at different N levels, βNRI was significantly and positively related to the differences in N across the low N range (r = 0.57; *P* < 0.0001), and decreased weakly with the differences in N across the high N range (r = −0.16; *P* = 0.001) (Fig. 4c). For comparisons within each N level, βNRI results (Fig. 4d) showed that as N deposition rate increased (0–5.25 g N m^−2^ yr^−1^), the role of homogeneous selection decreased rapidly across relatively deep phylogenetic distance (linear regression, R^2^ = 0.75; *P* < 0.0001), and all βNRI values for 0 g N m^−2^ yr^−1^ samples were <−2 (μ = −2.51 with σ = 0.26), representing a strong homogeneous selection. Although there was a significant regression relationship for higher N range (R^2^ = 0.29; *P* < 0.0001), all values were >−2 and <+2. The patterns of βNRI and βNTI values within each N level aligned well with those between N levels (Fig. 4). The βNRI patterns highlight a strong effect of N addition on microbial structure across deep phylogenetic relationships. However, the βNTI patterns show that the selection effect across fine phylogenetic relationships may be not related with N input directly.

### Ecological processes influencing shifts in phylogenetic structure of active microbes in response to moisture pulse

**Figure 5:**
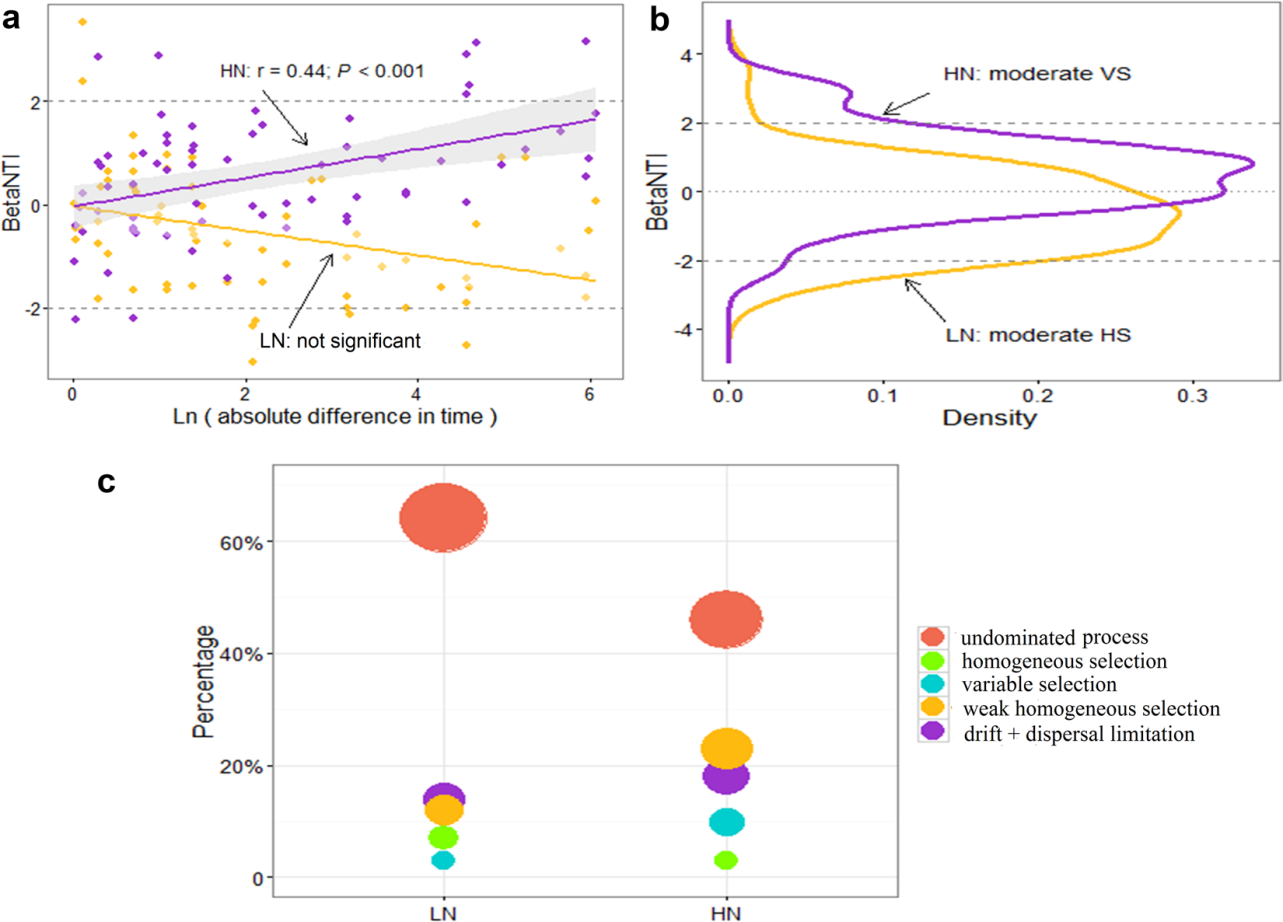
βNTI patterns from empirical comparisons (**a**) and simulated distributions (**b**), and fractions of ecological processes (**c**) in moisture pulse experiment. (**a**) βNTI values between different time points were fitted against Ln-transformed absolute differences in time. The statistics were from Mantel test (10000 permutations). Horizontal dashed lines represented values of +2 and −2 beyond which an individual β-deviation value was considered statistically significant. (**b**) βNTI distributions were obtained from kernel density estimates. (**c**) Bubble plot was used to show the fraction that selection, weak selection, drift coupled with dispersal limitation and the undominated process contribute to the community assembly. HS: homogeneous selection; VS: variable selection. LN: low nitrogen soil (N deposition rate: 5.25 g N m^−2^ yr^−1^); HN: high nitrogen soil (N deposition rate: 28 g N m^−2^ yr^−1^).

To evaluate the extent to which the profiles of active community members were influenced by moisture pulse, we first calculated the correlation between the βNTI distance matrix—based on rRNA sequencing—and Ln-transformed time. Mantel test (10000 permutations) indicated that there was significant correlation for HN (r = 0.44; *P* < 0.001, Fig. 5a) but not for LN (r = −0.38; *P* > 0.98), which suggested a selective pressure imposed by rewetting and drying on the HN samples. Permutational analysis of the multivariate dispersions (PERMDISP)^40^ method was used for the analysis of multivariate homogeneity of group dispersions. The mean of distance-to-centroid values of βNTI in HN was higher than that in LN (PERMDISP, *P* < 0.05), which indicated a large difference of the distributions of βNTI values between HN and LN. Density plots showed clear separation between these distributions (Fig. 5b). Higher βNTI mean value in HN (μ = 0.57 with σ = 1.17) suggested a stronger influence of variable selection than within LN (μ = −0.55 with σ = 1.22). In addition, more significant positive (>+2) βNTI values further suggested there was stronger variable selection in HN following the moisture pulse (Fig. 5a and c). We also used Bray-Curtis-based Raup-Crick (RC_bray_)^19^ to assess the community compositional turnover. The results of βNTI, together with RC_bray_, showed that variable selection governed 10% of temporal turnover in HN and 3% in LN. Homogeneous selection, dispersal limitation, and undominated processes governed 3%, 18%, and 46% of temporal turnover in HN and 7%, 14%, and 64% in LN (Fig. 5c). Our combined results showed that response to moisture perturbation was dependent on N addition history.

## Discussion

In this study, we first investigated the phylogenetic structure and turnover of prokaryotic communities along the N gradient based on the 16S rDNA sequencing. We examined phylogenetic signal of OTU-level niches to evaluate our ability to make ecological inferences from the phylogenetic patterns. We found that phylogenetic signals only occurred across short phylogenetic distance (Fig. 2), in line with previous studies on microbial communities^29,34,41^. There are few theoretical simulation studies focused on the relationship between phylogenetic signal and phylogenetic community structure such that we could not quantitatively compare the results from Mantel correlogram and those from Pagel’s λ. As such, we chose to examine both βNTI (which focuses on structure across short phylogenetic distances) and βNRI (which includes deep phylogenetic relationships) to investigate phylogenetic turnover, as has been done previously^38,39^.

We found that across relatively low N levels, the addition of N increased phylogenetic diversity (Fig. 3) and increased stochasticity when accounting for deep phylogenetic relationships, between and within N levels (Fig. 4). These outcomes are consistent with Chase’s^25^ finding that stochastic community assembly causes higher biodiversity in more productive environments. From a macro-ecological perspective, N fertilizer input, coupled with indirect changes of soil properties, might reduce competition by providing diverse resources and creating more niche dimensions for different guilds and may strengthen priority effects^42^ to sustain more species. For instance, the stimulation of plant growth by N inputs could increase aboveground litter inputs to soil and also belowground carbon inputs by increasing root growth and root exudates^43^. Control samples exhibited the strongest homogeneous selection across deep phylogenetic distances (Fig. 4d). This was concordant with the conclusion that emergence of stable environments can lead to stable levels of homogeneous selection^18^. Dini-Andreote *et al*.^20^ observed a negative relationship between resource supply and stochasticity. That may stem from a selection effect driven mainly by some physicochemical factors (e.g., salinity) or their interactions, accordant with the higher N gradient (5.25–28 g N m^−2^ yr^−1^) in our study. Therefore, explaining the underlying processes shaping community patterns can be closely related with the shifts of dominant factors.

The βNRI patterns suggest that the level of N deposition selects for specific, major clades, but the strength of selection for these clades decreases as N increases, up to about 5 g N m^−2^ yr^−1^. The selective environments did not change a lot at higher N levels. The βNTI results indicate that within these major clades taxa are likely being selected based on localized biotic/abiotic conditions that are unrelated to the level of N. From that perspective, the level of N provides an overarching selective pressure that applies across deep evolutionary relationships, and microscale conditions impose additional selective pressures to select specific taxa from within N-selected clades. That suggests different levels of selection. N has the primary effect, and other (unmeasured) variables appear to have secondary effects.

Although the influence of variable selection based on βNTI was relatively stable along the N gradient (Fig. 4a), the factors imposing selection might be different between the N range of 0–10.5 g N m^−2^ yr^−1^ and the range of 10.5–28 g N m^−2^ yr^−1^. For example, when N addition was larger than 10.5 g N m^−2^ yr^−1^, selection may be imposed by concomitant shifts in physicochemical conditions (e.g., low pH and high salinity^23^). This interpretation is consistent with previous work showing that a large fraction of stress-intolerant taxa disappear in high N soil (pH < 6)^23^ and that bacterial diversity decreases as soil pH drops below 6.5^44^. Low phylogenetic diversity in the high N community (e.g., 28 g N m^−2^ yr^−1^) further points towards a strong influence of soil physicochemical perturbations. These results emphasize that in microbial systems increased resource supply has a variety of consequences such that we cannot generally expect that the relative influence of stochasticity is to increase with resource supply^25^. In particular, a relationship between resource supply and stochasticity may only be expected when resource input does not result in harsh (i.e., highly selective) abiotic conditions.

In this study, we treated samples from each plot (5 m × 5 m) as one composite community, and were not able to evaluate the influences of spatial scale or fine-scale heterogeneity. Exploring the spatial hierarchical structure of microbial communities and associated ecological assembly processes would be an exciting extension of our work that would require additional null model formulations and a rigorous definition of local community^14,38,45,46^. In addition, although many studies have studied phylogenetic patterns of prokaryotes in various natural and artificial environments^47^, few elucidate shifts in assembly processes along gradients of long-term resource addition. We suggest that more manipulative field studies that impose these types of environmental gradients should be conducted toward understanding microbial community assembly at different spatial and taxonomical scales.

Using rRNA as the biomarker, we explored the response of phylogenetic structure to short-term moisture perturbation in terms of OTU metabolic activity. We viewed LN and HN as different regional species pools and studied their differences in temporal community turnover. Previous work has similarly studied the phylogenetic structure of resuscitated prokaryotes following a rewetting pulse by studying rRNA^26^.

Taking this approach first revealed phylogenetic signal in the temporal response of microbial taxa, which is consistent with previous work showing phylogenetic signal in organismal niches irrespective of ecosystem type^30,31,34,48^. Within the Mantel correlogram for HN, we observed, however, that phylogenetic signal was lost and then regained as increasingly longer phylogenetic distances were evaluated (Fig. 2b). A potential reason might be that strong niche partitioning was a dominant process across deep phylogenetic relationships in HN. For example, *Actinobacteria*, *Firmicutes*, and *Proteobacteria* in HN had more drastic changes in relative-abundances compared to LN (Fig. S1). These three phyla may be typical of endogenous succession—as discussed by Placella *et al*.^26^ and Fierer *et al*.^49^—and they exhibited more apparent niche differences in HN than in LN (Fig. S1). The significant negative correlations within the Mantel correlogram (especially in HN) might result from convergent evolution of lineages around intermediate phylogenetic distance. For instance, at the class level, the relative abundances of *Planctomycetia* (*Planctomycetes* phylum) and *Actinobacteria* (*Actinobacteria* phylum) both decreased rapidly after the first rewetting pulse (Fig. S2). This suggests that these two taxonomic groups may have similar temporal niches, which could have led to the negative correlations within the Mantel correlogram.

In addition to examining patterns in phylogenetic signal, we used null models to evaluate community assembly processes governing response to moisture perturbation, which revealed an important role of environmental history. In particular, under the same moisture perturbation, phylogenetic turnover of active microbial taxa within HN was more rapid than within LN (see also the OTU heatmap, Fig. S3). This historical effect appeared to lead to a trend towards variable selection in HN and a trend towards homogeneous selection in LN (Fig. 5b). Another study^30^ reports that, regardless of disturbance type, zooplankton communities already under stress (i.e., those in acidic lakes) changed most when disturbed. Such historical contingency in the response to perturbation in belowground systems is increasingly recognized^50–52^ and is clearly an important aspect of ecosystem function that requires mechanistic understanding.

Expanding the analyses to account for deep phylogenetic relationships revealed a strong influence of homogeneous selection, and this process may be especially influential under the HN conditions (Fig. S4). There was therefore stronger homogeneous selection across clades (βNRI) and stronger variable selection within clades (βNTI) in HN than LN. This suggests that certain clades were well adapted to high N conditions, but different taxa within each of those clades differed in their optimal moisture conditions. Thus, strong homogeneous selection in HN shown by βNRI might cause low phylogenetic diversity under a specific period of time.

Moderate homogeneous selection in LN across both short and deep phylogenetic distances might highlight the importance of niche overlap and metabolic coupling among species, which could be conducive to the stability of microbial communities^53^. Our results further showed that the undominated fraction was larger in LN than that in HN. This is consistent with Stegen *et al*.’s^19^ inference that the undominated fraction should increase with decreases in the strength of selection and/or with a shift toward moderate dispersal rates. The relatively large contribution of undominated process in LN might come from interactions among many ecological processes or factors, e.g. drift, biotic interactions, etc.^21^.

In summary, we elucidated how ecological community assembly processes shifted along an N addition gradient, and how the history of long-term N deposition influenced phylogenetic temporal turnover of microbial communities responding to pulse disturbance. Moderate N addition increased stochasticity across deep phylogenetic relationships and increased phylogenetic diversity. This positive effect on diversity was not maintained at higher levels of N addition, and we infer that too much N deposition can result in harsh environmental conditions (e.g., low pH), leading to the low phylogenetic diversity. We also found that while N level imposed a primary selective pressure, there were secondary selective pressures that were highly variable within and across levels of N addition. Our work also contributes to the growing body of literature showing that environmental history strongly influences response of microbial community to perturbation. Here we go beyond previous work to show that these historical contingencies apply to ecological assembly processes. Press disturbance, therefore, not only influences spatial turnover of communities by imposing different selection pressures, but also affects the response to pulse disturbance. Collectively, this study provides new insights into the complex connections among the history of environmental conditions, assembly processes, and microbial community dynamics. Elucidating these connections is necessary to understanding and ultimately predicting microbial response to perturbation.

## Methods

### Study site description and soil sampling

Soil samples were collected from a long-term N deposition experiment conducted at the Inner Mongolia Grassland Ecosystem Research Station (IMGERS, 116° 42′ E, 43° 38′ N and 1250 m a.s.l.). Detailed descriptions of the study site and sampling methods were provided in Bai *et al*.^54^ and Yao *et al*.^23^, respectively. Briefly the N deposition experiment was conducted using randomized block design, each level with six replicates. One plot was 5 × 5 m in size and separated by a 1-m buffer zone. Nutrients (pelletized NH_4_NO_3_ fertilizer) were uniformly applied to each plot with manual broadcasting in the mid-growing season (middle of June) annually beginning in 1999. There are six levels of nitrogen addition rates in this research (0, 1.75, 5.25, 10.5, 17.5 and 28 g N m^−2^ yr^−1^). Soil samples were collected from the top layer (depth: 0–10 cm; soil core diameter: 5 cm) in the middle of July 2012, about 1 month later after fertilization. Each sample was a mixture of ten individual soil cores along cross-line in one plot. In total we obtained 36 samples in the experimental field. Part of the soil was stored at −20 °C for molecular experiments, including DNA extraction and 16S rRNA gene amplicon sequencing (see Yao *et al*.,^23^ for this part and also the bioinformatics analysis). Other soil was air-dried to make the microbes dormant^55^, and sieved with 2 mm mesh to remove grass roots and stones. We stored the soil at 4 °C to reduce freeze-thaw disturbance^9^. Although soil properties might change to a certain degree at this temperature, we decided that this would not largely influence the explanations of ecological theories in this study. It has been shown that the resuscitation of prokaryotes under moisture pulse is strongly associated with microbial life-strategies^24,29^.

### Moisture pulse experiment

In 2014, we conducted the moisture pulse experiments using the air-dried samples. We used two levels of N treated soils, low N (LN, 5.25 g N m^−2^ yr^−1^) and high N (HN, 28 g N m^−2^ yr^−1^), for the rewetting experiment. Before rewetting treatment, replicate field samples were mixed equally and thoroughly into one composite sample for each N level to ensure the same initial community. Twenty grams of air-dried soil was weighted into a 300 mL glass bottle with a rubber stopper. For each soil, three bottles were allocated for destructive sampling at every time point. On the first day, wetting was initiated by adding 10 mL of ultra-pure water using a disposable syringe, approximately equivalent to a 35-mm rainfall event being distributed. Drying of the soils was achieved by adding 10 g of desiccant allochronic silica gel (chromatography grade, Yurui Ltd, Qingdao, China) wrapped by a monolayer sterile absorbent gauze on the second day^55^. The silica gel was replaced each day. The columnar silica gel was kept hanging using thin yarn to ensure that the silica gel did not directly contact the soil. The total incubation time (determined by the pre-experiment) was about 6 days for single rewetting and drying treatment. The second rewetting was performed two days later after the first wetting-drying cycle (Fig. 1). Soils in the bottles were destructively sampled at 30 min, 2 h, 8 h, 1 d, 2 d, 3 d, 4 d and 6 d (T1–T8) after the first rewetting, and at 30 min, 2 h, 8 h, 1 d (2T1–2T4) after the second rewetting. The bottles were incubated at 25 °C. Gravimetric moisture content was measured by weighing soil samples before and after oven-drying at 105 °C for 24 h.

### Molecular methods and sequencing data analysis

Total RNA of the soil sample from the moisture pulse experiment was extracted using the Omega D5625-01 soil RNA kit (Omega Bio-Tek, USA) according to the manufacturer’s instructions. The cDNA was generated from 100 ng of the extracted RNA for each sample using the Reverse Transcription Kit (Thermo Scientific, USA) and stored at −40 °C for downstream use. Universal primer 515 F (5′-GTGCCAGCMGCCGCGGTAA-3′) and 806 R (5′-GGACTACHVGGGTWTCTAAT-3′) with 12 nt unique barcode, was used to amplify the V4 hypervariable region of 16S rRNA gene^56^. The PCR mixture (25 μl) contained 1x PCR buffer, 1.5 mM MgCl_2_, each deoxynucleoside triphosphate at 0.4 μM, each primer at 1.0 μM, 0.5 U of Ex Taq (TaKaRa, Dalian, China) and 1 μl cDNA. The PCR amplification program included initial denaturation at 94 °C for 3 min, followed by 30 cycles (94 °C for 20 s, 56 °C for 30 s, and 72 °C for 45 s), and a final extension at 72 °C for 10 min^57^. Triplicate PCR reactions were conducted for each sample, and PCR products were pooled for purification with electrophoresis and OMEGA Gel Extraction Kit (Omega Bio-Tek, USA). PCR products from different samples were pooled with equal molar amount, and then applied to paired-end sequencing (2 × 250 bp) with the Illumina Miseq sequencer at Chengdu Institute of Biology, Chinese Academy of Sciences.

The sequencing data were processed using QIIME Pipeline–Version 1.7.0^58^. All reads were trimmed and assigned to each sample based on the barcodes. The sequences with high quality (length > 200 bp, without ambiguous base ‘N’, average base quality score > 30) were extracted and further screened for chimera checking using the Uchime algorithm^59^. Then, all the samples were randomly resampled to 9000 reads. Sequences were clustered into operational taxonomic units (OTUs) at the 97% identity threshold. Taxonomy was assigned using the greengenes data sets. Phylogenetic maximum likelihood–approximation tree was reconstructed using the generalized time-reversible (GTR) model in FastTree 2.1.1^60^. We excluded rare OTUs (occurred in less than three replicate samples for 16S rRNA-data; the average abundance lower than 0.01% for N deposition experiment) to reduce noise.

### Analytical framework for community data

We used phylogenetic null models to infer ecological assembly processes, which requires significant phylogenetic signal in organismal ecological niches^29^. Although the microbial niche is multidimensional, there was strong collinearity among several important environmental factors^23^. We therefore use the amount of N addition to evaluate phylogenetic signal in relation to our analyses of spatial turnover in community composition. The optimal N level for each taxa was estimated using the abundance-weighted approach used in previous studies^18,34^. For analyses of temporal turnover in the active community, we evaluated phylogenetic signal using time as the niche axis. We specifically used eight time points after the first rewetting, representing a whole rewetting period (Fig. 1), to test phylogenetic signal. In the pulse experiment, we viewed LN and HN as different species pools, so the abundance-weighted mean time value was obtained for each OTU in LN and HN, separately. We calculated Pagel’s λ^32^ with the function ‘phylosig’ in R package *phytools* ^61^. We also performed Mantel correlograms using ‘mantel.correlog’ in package *vegan* ^62^. The Euclidean distances of OTU-specific niche values and Patristic distances were used^63,64^. We partitioned the phylogenetic distance into classes by 0.02 units directionally from tip to root of the phylogenetic tree after standardization^34^.

Phylogenetic diversity (PD) and standardized effect size of PD (ses.PD) were calculated with package *picante*. Observed OTU number and the corresponding phylogenetic diversity can be affected largely by the sampling effects and sequencing depth. The data filtering can also influence such calculations. Here, we used standardized effect size instead of resampling on sequences or the OTU table to reduce the sampling and sequencing effects on the observed phylogenetic diversity^14^. For beta diversity, we first adopted the beta mean nearest taxon distance (βMNTD) along N gradient^29^. According to the definition, βMNTD is a ‘terminal’ metric of phylogenetic β-diversity that quantify phylogenetic turnover based on the phylogenetic distance among the closest relatives. Standardized effect size (beta nearest taxon index, βNTI) was obtained by using null model to infer the relative importance of deterministic processes in generating β-patterns^65^. For this reason, we randomized the phylogenetic relatedness of species in the whole N deposition data set (1000 runs). This shuffling-approach can fix the observed levels of species α-diversity and β-diversity^66^.

N input could cause many indirect effects on prokaryotic communities because of the fluctuations of soil properties. For example, soil pH could have a large effects on communities at a relatively high taxonomic level^23^. Thus, to fully explore phylogenetic turnover to see whether there was a turnover pattern around deep phylogenetic distance, we also calculated beta mean pairwise phylogenetic distance (βMPD) and its standardized effect size (beta net relatedness index, βNRI), which are the’basal’ metrics of phylogenetic β-diversity^67^. In this research, we defined the phylogenetic turnover as the turnover of lineages caused by species replacement. Unifrac^68^, which refers to both shallow and deep phylogeny and is tightly related to phylogenetic diversity, may be futile for detecting the patterns abovementioned and was not adopted for this study (also see the discussion in supplementary information)^69^. βNTI, together with βNRI, can help us better infer the relationships among N niche conservatism, phylogenetic hierarchical structure, and species turnover^39^. All the calculations of observed β-diversity and β-deviations were weighted by species abundance. Within each N level, the environmental heterogeneity induced by N input could also produce differences (niche-selection) among microbial communities, so we further use βNTI and βNRI to quantify the turnover among communities within each N level. βNTI and βNRI were calculated via R scripts provided in supplemental material.

To quantify phylogenetic temporal turnover of communities under pulse disturbance, we also calculated βNTI between paired communities at different time points based on rRNA data. Because our goal was to compare the turnover between LN and HN under short-term disturbance, we treated LN and HN as different species pools and carried out the randomizations of null models for LN and HN separately. To test if the mean βNTI within each group was significantly different from the expected value of zero^29^, we employed the non-parametric permutation test based on the Monte Carlo simulation (10000 runs), and we also used this method to test the differences between LN and HN in dispersion of βNTI using distance-to-centroid values (permutational analysis of multivariate dispersions, PERMDISP^70^) obtained by the ‘betadisper’ in *vegan*. In our data sets, the average distance-to-centroids and average paired-dissimilarities showed the same pattern, so we used scatter and density plots to present the summary statistics^71^. In further analysis we used the Mantel test to calculate the correlation between time factor and βNTI. Thus, we could evaluate the differences of deterministic effects on phylogenetic turnover between LN and HN. The sampling time was Ln-transformed according to the sampling regime. The relative abundances of different taxa along the temporal gradient were fitted using locally weighted polynomial regression to show the tendency after rewetting.

To quantify the compositional turnover in LN and HN, we used a modified Raup–Crick metric (Bray-Curtis-based Raup-Crick, RC_bray_) to assess whether the compositional turnover was governed primarily by drift^18,72^. We applied null model to simulate species distribution by randomly sampling individuals from each species pool with preserving species occurrence frequency and sample species richness. In the pulse experiment, we incubated each bottle separately. So there was no effect of dispersal among communities. We inferred an influence of ‘weak homogeneous selection’ when compositional turnover was less than expected based on RC_bray_, but phylogenetic turnover did not deviate significantly from a stochastic expectation. The fraction of pairwise comparisons with |βNTI| < 2 and RC_bray_ <−0.95 provided an estimate of weak homogeneous selection. Weak homogeneous selection might come from environmental conditions that impose a weak strength of selection and/or opposing selection pressures.

There was no dispersal among samples during the pulse disturbance experiment. This provides a situation in which stochastic ecological drift may lead to significant divergence in community composition within and across time points. The fraction of pairwise comparisons with |βNTI| < 2 and RC_bray_ >  + 0.95 was used as an estimate of the relative influence of ecological drift coupled with dispersal limitation. Further, the fraction of pairwise comparisons with |βNTI| < 2 and |RC_bray_| < 0.95 estimated the amount of turnover in community composition that was ‘undominated’ by any single process^19^. All statistical analyses were performed in R 3.0.1^73^.

### Nucleotide sequence accession number

Raw sequence data have been submitted to the NCBI Sequence Read Archive (SRA) (http://trace.ncbi.nlm.nih.gov/Traces/sra/sra.cgi) under accession number SRR5678524.

## Electronic supplementary material

Supplementary information
