# Supplementary material for: Long-term nitrogen addition affects the phylogenetic turnover of soil microbial community responding to moisture pulse
Source: Sci Rep. 2017 Dec 13;7:17492. doi: 10.1038/s41598-017-17736-w (PMC5727477; doi:10.1038/s41598-017-17736-w)
Supplement: Supplementary file 1 — Supplementary information [file 41598_2017_17736_MOESM1_ESM.doc]

**Supporting Information:**

**Long-term nitrogen addition affects the phylogenetic turnover of soil microbial community responding to moisture pulse**

Chi Liu,^1^ Minjie Yao,^1*^ James C. Stegen,^2^ Junpeng Rui,^1^ Jiabao Li^1^, Xiangzhen Li^3*^

1*Key Labor­atory of Environmental and Applied Microbiology, Chinese Academy of Sciences; Environmental Microbiology Key Laboratory of Sichuan Province, Chengdu Institute of Biology, Chinese Academy of Sciences, Sichuan 610041, China*

*2 Earth and Biological Sciences Directorate, Biological Sciences Division, Pacific Northwest National Laboratory, Richland, WA 99352, USA*

*3 Fujian Provincial Key Laboratory of Soil Environmental Health and Regulation, College of Resources and Environment, Fujian Agriculture and Forestry University, Fuzhou 350002, China*

^*^ Corresponding authors: Xiangzhen Li or Minjie Yao

E-mail: [lixz@cib.ac.cn](mailto:lixz@cib.ac.cn) or [yaomj@cib.ac.cn](mailto:yaomj@cib.ac.cn)


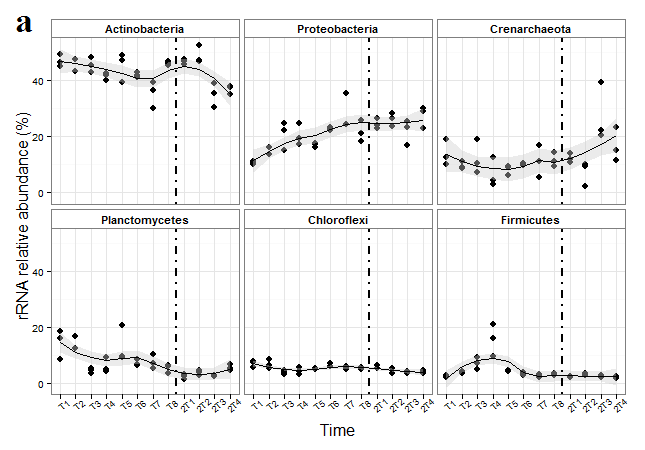

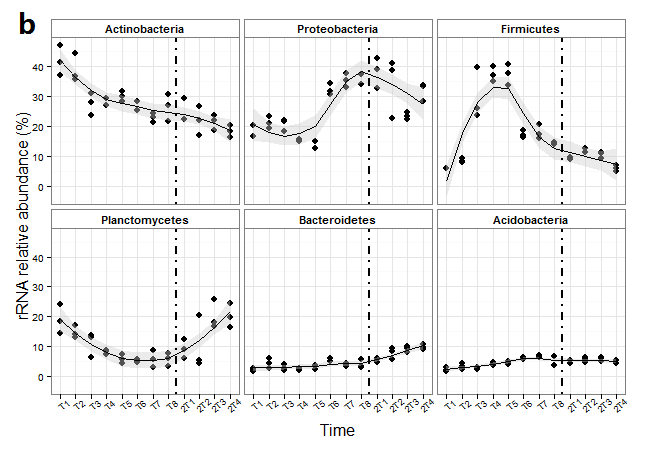


**Fig. S1.** Six most abundant phyla were exhibited for both LN (a) and HN (b) based on rRNA analysis. Although the data belong to the time series, the discrete time axis was chosen arbitrarily to better show the relative abundance tendency for each taxa. In each panel, two parts, divided by the two-dash line, denoted the sampling points after the first and second rewetting, respectively. T1-T8: 0.5, 2, 8, 24, 48, 72, 96, 144 (h); 2T1-2T4: 0.5, 2, 8, 24 (h).


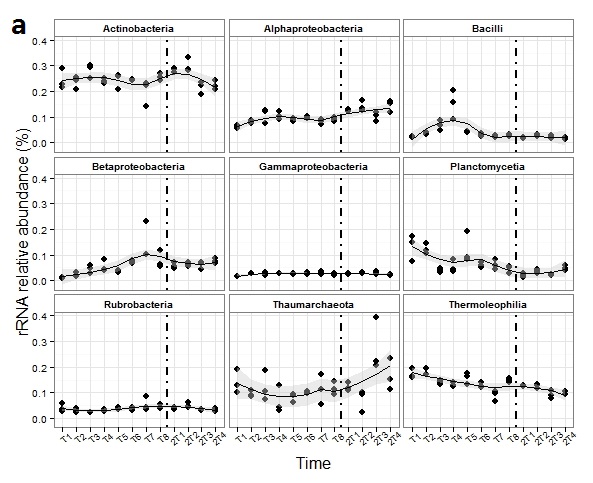


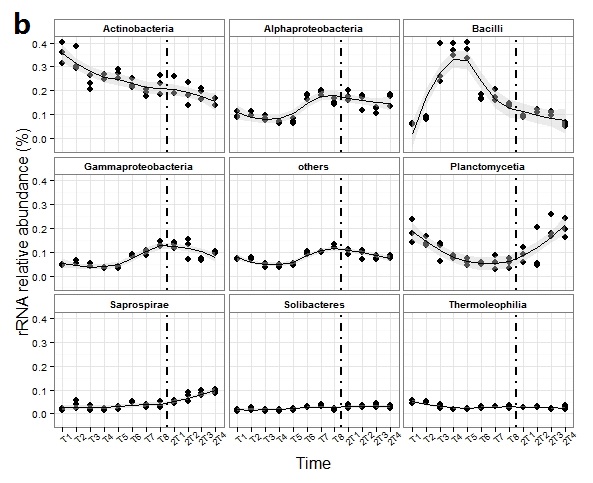


**Fig. S2.** Nine most abundant classes (in HN, ‘others’ represents the sum of the abundances of all unclassified classes) were exhibited for both LN(a) and HN(b) based on rRNA analysis. In each panel, two parts, divided by the two-dash line, denoted the sampling points at the first and second rewetting, respectively. T1-T8: 0.5, 2, 8, 24, 48, 72, 96, 144 (h); 2T1-2T4: 0.5, 2, 8, 24 (h).


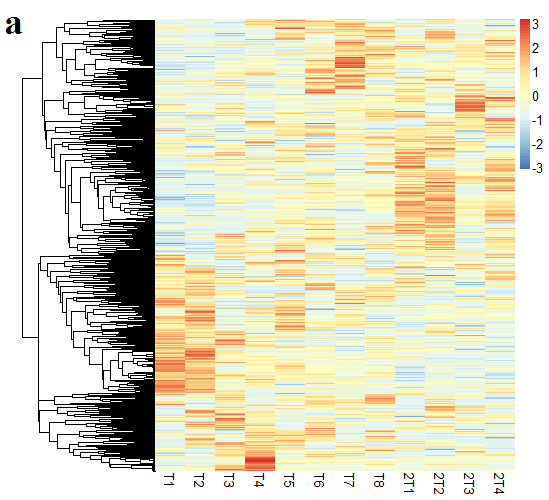


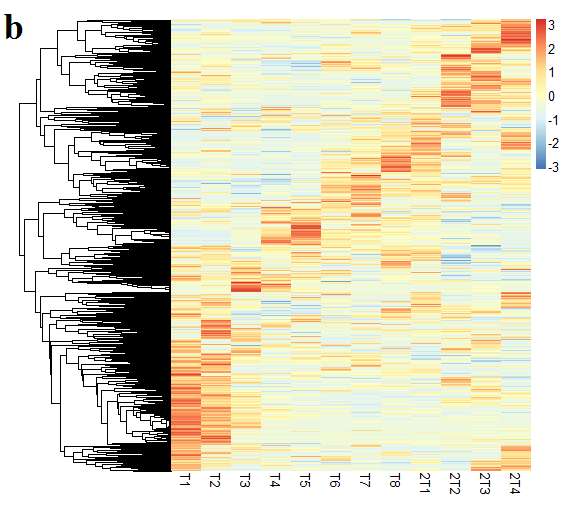


**Fig. S3.** Heatmap of OTU abundance for both LN (a) and HN (b) based on rRNA analysis. The Euclidean distance between OTUs and the average clustering method were adopted. T1-T8: 0.5, 2, 8, 24, 48, 72, 96, 144 (h); 2T1-2T4: 0.5, 2, 8, 24 (h).

**
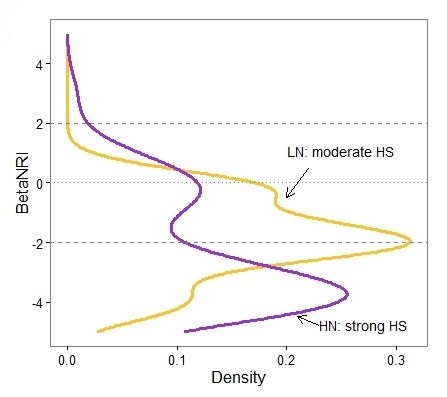
**

**Fig. S4.** βNRI density plot using rRNA dataset in moisture experiment. Horizontal dashed lines denoted values of +2 and -2, the values beyond which an individual β-deviation value was considered statistically significant. HS: homogeneous selection; VS: variable selection; LN: low nitrogen soil (N deposition rate: 5.25 g N m^-2^ yr^-1^); HN: high nitrogen soil (28 g N m^-2^ yr^-1^).

**
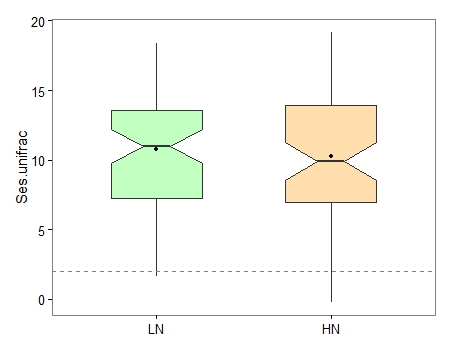
**

**Fig. S5.** The pattern of the β-deviation for Unifrac in moisture pulse experiment. LN: low nitrogen soil (N deposition rate: 5.25 g N m^-2^ yr^-1^); HN: high nitrogen soil (28 g N m^-2^ yr^-1^). Horizontal dashed line denoted values of +2, the value beyond which an individual β-deviation value was considered statistically significant.

**Relevant discussion:** The metric Unifrac (Lozupone and Knight, 2005) is commonly used to measure community phylogenetic dissimilarities in microbial ecology, such as exploring the distance-decay patterns across space and time. Observed patterns of β-diversity and the underlying mechanisms can be strongly influenced by stochastic sampling effects (Chase and Myers, 2011). We did not find a different turnover pattern between HN and LN based on the Unifrac algorithm after controlling sampling effects **(Fig. S5)**. We also found that the mean ses.unifrac were both significantly larger than +2 for HN and LN. Such scenario was largely different with the patterns shown by βNTI and βNRI. We speculated that this algorithm might be not suitable for directly comparing the differences of phylogenetic turnover patterns between different regions with distinct diversity, because Unifrac utilizes the basal parts of the phylogeny and can be separated into two components accounting for ‘true’ turnover of lineages and phylogenetic diversity gradients (see calculations in Leprieur *et al*., 2012). Explaining the details of the algorithm-difference between Unifrac and other beta-metrics was beyond the scope of this study. Overall, beta-metrics were critical for explaining beta-diversity patterns.

**References:**

Chase, J.M., Myers, J.A., 2011. Disentangling the importance of ecological niches from stochastic processes across scales. Philosophical Transactions of the Royal Society of London Series B - Biological Sciences 366, 2351-2363.

Leprieur, F., Albouy, C., De Bortoli, J., Cowman, P.F., Bellwood, D.R., Mouillot, D., 2012. Quantifying phylogenetic beta diversity: distinguishing between ‘true’turnover of lineages and phylogenetic diversity gradients. PloS One 7, e42760.

Lozupone, C., Knight, R., 2005. UniFrac: a new phylogenetic method for comparing microbial communities. Applied and Environmental Microbiology 71, 8228-8235.

**# R scripts for calculating betaNRI and betaNTI**

#First of all, check the OTU names in the OTU table and tip labels in the tree and #make these two match!

ses_betampd <- function(samp, tre, runs=1000, abundance.weighted = TRUE, iterations = 1000) {

# samp: OTU table, colnames are OTU id

library(picante)

# specify seeds for random number

set.seed(1234)

dis <- cophenetic(tre)

betaobs <- comdist(samp, dis, abundance.weighted)

N <- dim(samp)[1]

sesbeta_matrix <- matrix(nrow = N, ncol = N)

rownames(sesbeta_matrix) <- colnames(sesbeta_matrix) <- rownames(samp)

betaobs_vec <- as.vector(betaobs)

betampd_rand <- replicate(runs, comdist(samp, taxaShuffle(dis), abundance.weighted))

betampd_rand_mean <- apply(X = betampd_rand, MARGIN = 1, FUN = mean,

na.rm = TRUE)

betampd_rand_sd <- apply(X = betampd_rand, MARGIN = 1, FUN = sd,

na.rm = TRUE)

beta_obs_z <- (betaobs_vec - betampd_rand_mean)/betampd_rand_sd

for (i in 1:(N - 1)) {

x <- 1

for (j in (i + 1):N) {

sesbeta_matrix[j,i] <- beta_obs_z[x]

x <- x+1

}

beta_obs_z <- beta_obs_z[-c(1:(N-i))]

}

return(as.matrix(as.dist(sesbeta_matrix)))

}

ses_betamntd <- function(samp, tre, runs=1000, abundance.weighted = TRUE, iterations = 1000) {

# samp: OTU table, colnames are OTU id

library(picante)

set.seed(1234)

dis <- cophenetic(tre)

betaobs <- comdistnt(samp, dis, abundance.weighted)

N <- dim(samp)[1]

sesbeta_matrix <- matrix(nrow = N, ncol = N)

rownames(sesbeta_matrix) <- colnames(sesbeta_matrix) <- rownames(samp)

betaobs_vec <- as.vector(betaobs)

betamntd_rand <- replicate(runs, comdistnt(samp, taxaShuffle(dis), abundance.weighted))

betamntd_rand_mean <- apply(X = betamntd_rand, MARGIN = 1, FUN = mean,

na.rm = TRUE)

betamntd_rand_sd <- apply(X = betamntd_rand, MARGIN = 1, FUN = sd,

na.rm = TRUE)

beta_obs_z <- (betaobs_vec - betamntd_rand_mean)/betamntd_rand_sd

for (i in 1:(N - 1)) {

x <- 1

for (j in (i + 1):N) {

sesbeta_matrix[j,i] <- beta_obs_z[x]

x <- x+1

}

beta_obs_z <- beta_obs_z[-c(1:(N-i))]

}

return(as.matrix(as.dist(sesbeta_matrix)))

}

**# R scripts for calculating RC_bray_**

SES <- function(x) {

return((sum(x > x[length(x)]))/length(x))

}

rcbray <- function(samp, runs=1000) {

# samp:OTU table, colnames are OTU id

library(vegan)

library(picante)

set.seed(1234)

betaobs <- vegdist(samp, method="bray")

betaobs_vec <- as.vector(betaobs)

N <- dim(samp)[1]

sesbeta_matrix <- matrix(nrow = N, ncol = N)

beta_rand <- replicate(runs, vegdist(randomizeMatrix(samp, "independentswap"), "bray"))

beta_rand <- as.data.frame(beta_rand)

beta_rand[ ,(runs + 1)] <- betaobs_vec

beta_obs_z <- apply(X = beta_rand, MARGIN = 1, FUN = SES)

beta_obs_z <- (beta_obs_z - 0.5) * 2

for (i in 1:(N - 1)) {

x <- 1

for (j in (i + 1):N) {

sesbeta_matrix[j,i] <- beta_obs_z[x]

x <- x+1

}

beta_obs_z <- beta_obs_z[-c(1:(N-i))]

}

return(as.matrix(as.dist(sesbeta_matrix)))

}
